# Can Platforms Affect the Safety and Efficacy of Drug-Eluting Stents in the Era of Biodegradable Polymers?: A Meta-Analysis of 34,850 Randomized Individuals

**DOI:** 10.1371/journal.pone.0151259

**Published:** 2016-03-31

**Authors:** Yun-Feng Yan, Long Jiang, Ming-Duo Zhang, Xin-He Li, Mao-Xiao Nie, Ting-Ting Feng, Xin Zhao, Lu-Ya Wang, Quan-Ming Zhao

**Affiliations:** 1 Beijing Anzhen Hospital, Affiliated to Capital Medical University, Beijing Institute of Heart, Lung and Blood Vessel Diseases, The Key Laboratory of Remodelling-related Cardiovascular Diseases, Ministry of Education, Department of Cardiology, 2 Anzhen Road, Chaoyang District, Beijing, 100029, China; 2 Beijing Anzhen Hospital, Affiliated to Capital Medical University, Beijing Institute of Heart, Lung and Blood Vessel Diseases, The Key Laboratory of Remodelling-related Cardiovascular Diseases, Ministry of Education, Department of Atherosclerosis, 2 Anzhen Road, Chaoyang District, Beijing, 100029, China; University of Tampere, FINLAND

## Abstract

**Objective:**

In the era of bare metal stents (BMSs), alloys have been considered to be better materials for stent design than stainless steel. In the era of biodegradable polymer drug-eluting stents (BP-DESs), the safety and efficacy of BP-DESs with different metal platforms (stainless steel or alloys) have not yet been reported, although their polymers are eventually absorbed, and only the metal platforms remain in the body. This study sought to determine the clinical safety and efficacy of BP-DESs with different platforms compared with other stents (other DESs and BMSs).

**Methods:**

PubMed, Embase and Clinical Trials.gov were searched for randomized controlled trials (RCTs) that compared BP-DESs with other stents. After performing pooled analysis of BP-DESs and other stents, we performed a subgroup analysis using two classification methods: stent platform and follow-up time. The study characteristics, patient characteristics and clinical outcomes were abstracted.

**Results:**

Forty RCTs (49 studies) comprising 34,850 patients were included. Biodegradable polymer stainless drug-eluting stents (BP-stainless DESs) were superior to the other stents [mainly stainless drug-eluting stents (DESs)] in terms of pooled definite/probable stent thrombosis (ST) (OR [95% CI] = 0.76[0.61–0.95], p = 0.02), long-term definite/probable ST (OR [95% CI] = 0.73[0.57–0.94], p = 0.01), very late definite/probable ST (OR [95% CI] = 0.56[0.33–0.93], p = 0.03) and long-term definite ST. BP-stainless DESs had lower rates of pooled, mid-term and long-term target vessel revascularization (TVR) and target lesion revascularization (TLR) than the other stainless DESs and BMSs. Furthermore, BP-stainless DESs were associated with lower rates of long-term death than other stainless DESs and lower rates of mid-term myocardial infarction than BMSs. However, only the mid-term and long-term TVR rates were superior in BP-alloy DESs compared with the other stents.

**Conclusion:**

Our results indirectly suggest that BP-stainless DESs may offer more benefits than BP-alloy DESs in the era of BP-DESs. Further well-designed RCTs comparing BP-stainless with BP-alloy DESs are needed to confirm which platform is better.

## Introduction

The use of first-generation drug-eluting stents (DESs) has significantly reduced the risk of restenosis and repeat revascularisation compared with the use of bare-metal stents (BMSs).[1,2]. However, concerns have been raised over the increased risk of stent thrombosis (ST) attributed to the chronic inflammatory response triggered by remnants of the polymer material.[3,4] Therefore, DESs with biocompatible durable polymers (DP), polymer free (PF) DESs and DESs with biodegradable polymers (BP) that are eventually absorbed have been developed to lower the risk of inflammation and ST. These new BP-DESs have been thoroughly investigated in clinical trials and have been shown to achieve acceptable safety and efficacy outcomes.[5–9]

Drug-eluting stents are composed of a metal platform, antiproliferative drugs and polymers that control the release of the drugs. Regarding the platform, stainless steel and alloys are both widely used. In the era of BMSs, alloys, such as cobalt–chromium, have been shown to have superior strength compared with steel and are biologically inert; therefore, alloys might replace steel as the material of choice for stent design.[10] However, regarding BP-DESs, although the polymers are eventually absorbed so that only metal platforms remain in the body, the clinical safety and efficacy of BP-DESs with different platforms have not been clearly demonstrated. Studies of BP-DESs with different metal platforms have obtained conflicting results. One network meta-analysis[11] has shown that stainless BP biolimus-eluting stents (BESs, Biomatrix and Nobori) are associated with a higher rate of definite/probable ST than cobalt–chromium everolimus-eluting stents (CoCr-EES) (Abbott Vascular and Boston Scientific). In contrast, the SORT OUT VI[12] and BIOSCIENCE[13] studies comparing BP-DESs with different platforms (stainless steel and alloy) to cobalt–chromium DESs have found no significant differences in ST. Accordingly, we performed a meta-analysis to determine the clinical safety and efficacy outcomes of BP-DESs with different platforms by artificially dividing the BP-DESs into BP-stainless DESs and BP-alloy DESs.

## Materials and Methods

The Preferred Reporting Items for Systematic Reviews and Meta-Analyses (PRISMA) statement[14] was strictly followed in the present meta-analysis **(**S1 PRISMA Checklist). Because we extracted patient data from original articles, no ethical approval was needed for this meta-analysis.

### Search strategy

We searched the PubMed, Embase and Clinical Trials.gov computerized databases for randomized clinical trials uploaded through June 2015 in humans comparing biodegradable polymer stents with other types of stents **(**S1 Table). To improve the recall ratio, a manual search of the reference lists of the included articles, editorials, recent reviews and meta-analyses was also performed.

### Study selection

Our meta-analysis included only randomized controlled trials (RCTs) in humans comparing BP-DESs with other stents. The type of control stent used could not be a bioresorbable vascular scaffold or another BP-DES. The control stents used included DP-DESs, PF-DESs and BMSs. The exclusion criteria included the following: (i): optical coherence tomography (OCT) studies, intravascular ultrasound (IVUS) studies and conference papers in which the clinical data of interest could not be extracted; (ii): sub-group analyses, pooled analyses or post-hoc analyses in which the data were incomplete or repetitive; (iii): articles in which the comparison of implanted DES types was not designed in advance, and the investigators arbitrarily selected the type of control stents (for example, BP-DESs vs. any DESs); (iv): clinical trials with a follow-up period that was not one year or equivalent to the longest follow-up period. For example, for the LEADERS trial, the longest follow-up period was 5 years, so the two-year follow-up results were excluded; and (v): articles that were not published in English or Chinese. Our search placed no restrictions on the length of follow-up, sample size, patient or lesion characteristics or publication state.

### Data extraction

Two independent researchers (Zhang Mingduo and Li Xinhe) reviewed the abstracts and full texts of the relevant articles to assess their eligibility. Two researchers (Nie Maoxiao and Feng Tingting) collected the information regarding the study design, stent types compared, sample size, primary endpoint, major inclusion criteria, maximal length of follow-up, duration of DAPT, article language, baseline patient characteristics (age, percentage of males, diabetes, hypertension, hyperlipidemia, prior myocardial infarction (MI) and prior percutaneous coronary intervention (PCI)), data to assess the risk of bias and data of the endpoints of interest. The authors discussed the articles with additional data or unclear data.

### Risk of bias assessment

The Cochrane Collaboration’s tool for assessing the risk of bias[15] was used to evaluate the quality of the included RCTs. In summary, we assessed the selection, performance, detection, attrition and other biases. The assessment was conducted independently by two researchers (Li Xinhe and Zhang Mingduo), and conflicts were resolved by discussion.

### Endpoint assessment

We predefined definite/probable stent thrombosis (ST) of the pooled (the longest follow-up period) analysis as the primary safety endpoint. Other safety clinical endpoints included definite ST, death by any cause, cardiac death and myocardial infarction (MI). Target lesion revascularization (TLR) and target vessel revascularization (TVR) were predefined efficacy endpoints. All endpoints were defined according to the Academic Research Consortium (ARC) guidelines[16]. For each endpoint, we extracted the most inclusive definitions whenever possible; for example, all TVR instead of clinically or ischemia-driven TVR was extracted.

### Grouping

After the pooled analysis of BP-DESs and other stents, we performed our subgroup analysis using the following two classification methods: stent platform and follow-up time. Based on the stent platform classification, the case group (BP-DESs) was divided into two subgroups: BP-stainless DESs and BP-alloy DESs. The control group (other stents) were divided into three subgroups: other stainless DESs, other alloy DESs and BMSs. Based on the follow-up time point of DESs implantation, pooled (maximum length of follow up), short-term (within 30 days), mid-term (within 1 year) and long-term (beyond 1 year) safety and efficacy endpoints were assessed. Regarding ST, the ARC recommended temporal categories were evaluated: [early ST (within 30 days), late ST (>30 days-1 year), very late ST (>1 year but excluding data within 1 year), acute ST (within 24 h), subacute ST (>24 hours to 30 days)] and composite definitions [mid-term ST (within 1 year), long-term ST (beyond 1 year)]. Subgroup analyses were performed for each stent platform in the different time categories; for example, BP-stainless DESs were compared with other stainless DESs in pooled, short-term, mid-term and long-term analyses.

### Statistical analyses

Odds ratios (ORs) and 95% confidence intervals (CIs) were calculated for each endpoint. The Cochran Q statistic and the I^2^ test were used to evaluate the effects of heterogeneity. To minimize the effects of heterogeneity among the groups, we applied only a random-effect model using the method of DerSimonian & Laird. The sensitivity analysis was conducted in two ways. One method was performed by removing individual studies sequentially and examining whether the results changed. Another way was to examine whether the significance of pooled endpoints was altered when we excluded articles published in Chinese. A visual inspection of funnel plots was applied to assess publication bias. All statistical analyses were conducted using Review Manager (version 5.3). P values < 0.05 were considered statistically significant.

## Results

### Study selection

**Fig 1:**
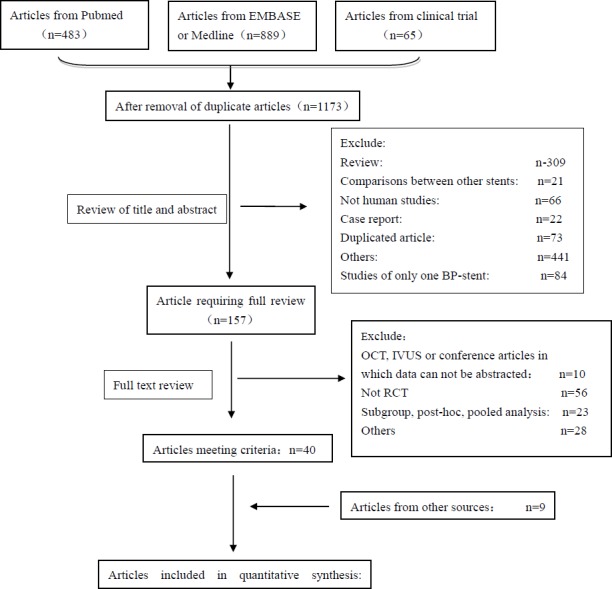
Flow diagram of study selection.

Fig 1 is a flow diagram of this meta-analysis. After removing duplicate reports, a total of 1173 relevant studies were identified. Finally, 40 clinical trials (49 studies) with 34850 patients were included in our analysis.

### Study and patient characteristics

The key characteristics of the included trials are shown in S2 Table. One article was published in Chinese, and the other articles were published in English. Six trials presented in 10 articles had a three-arm design. The median follow-up period was 19.6 months, with a range of 6 months to 5 years. The duration of dual anti-platelet therapy (DAPT) ranged from 3 months to ≥12 months (3 months: 2 trials, 6 months: 14 trials, 12 months: 22 trials, unclear: 2 trials). Twenty-six trials compared BP-stainless DES with other stents (10 alloy DESs, 6 BMSs, and 14 stainless DESs, of which 10 were first-generation DESs). Sixteen trials compared BP-alloy DESs to other stents (3 stainless DESs, 9 alloy DESs, and 2 BMSs). The features of the included patients are described in S3 Table.

### Risk of bias

As suggested by the Cochrane Collaboration’s tool, each item was defined as having low bias, high bias or unclear bias. The risk of bias graph is shown in S1 Fig.

### The primary endpoint: definite/probable ST

**Fig 2:**
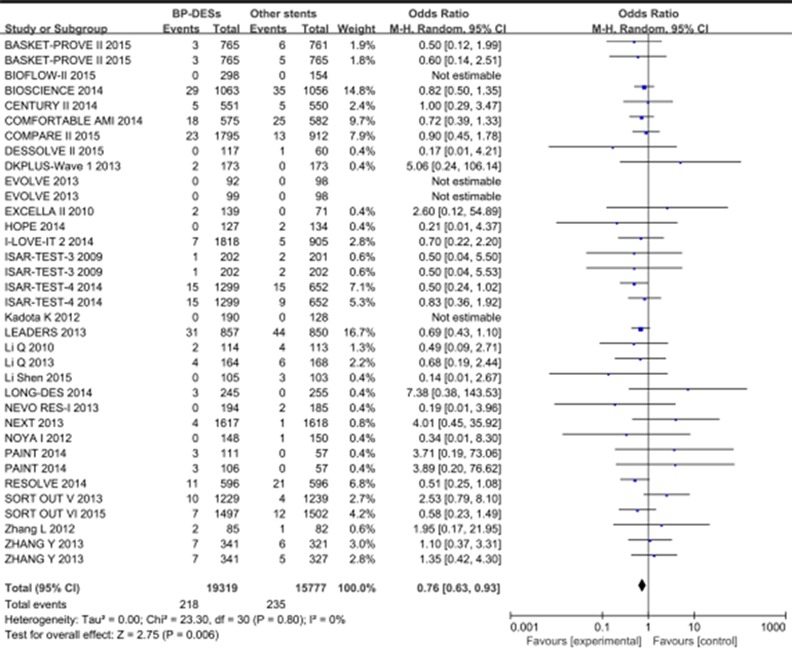
Pooled definite/probable ST of BP-DESs and other stents.

The primary safety endpoints of pooled definite/probable ST were presented in 29 trials with 35096 patients. **(**S4 Table**).** The risk of definite/probable ST was significantly lower in BP-DESs than in the other stents (OR [95% CI] = 0.76[0.63–0.93], p = 0.006; Fig 2**).**

#### BP-stainless DESs versus other stents

**Fig 3:**
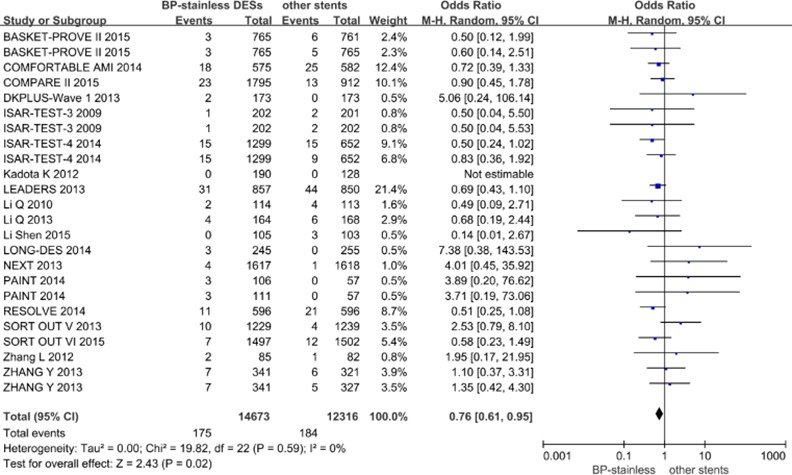
Pooled definite/probable ST of BP-stainless DESs versus other stents.

Nineteen studies with 26989 patients were included in the pooled analysis of definite/probable ST with BP-stainless DESs and other stents, and a statistically significant difference was observed between these two groups (OR [95% CI] = 0.76[0.61–0.95], p = 0.02; Fig 3). The same difference was observed in long-term ST (OR [95% CI] = 0.73[0.57–0.94], p = 0.01) and very late ST (OR [95% CI] = 0.56[0.33–0.93], p = 0.03). Regarding the other subgroups, BP-stainless was superior to other stainless DESs in very late ST (OR [95% CI] = 0.29[0.14–0.58], p = 0.0005) and long-term ST (OR [95% CI] = 0.70[0.50–0.98], p = 0.04) and was superior to other alloy DESs in late ST (OR [95% CI] = 0.46 [0.22–0.98], p = 0.04). No other significant differences were observed between the other subgroups.

#### BP-alloy DESs versus other stents

**Fig 4:**
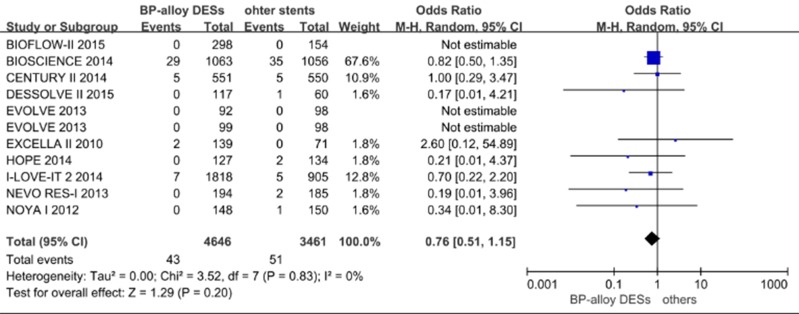
Pooled definite/probable ST of BP-alloy DESs versus other stents.

Comparisons of BP-alloy stents to other stents in terms of definite/probable ST were available in 10 studies with 8107 patients, and the pooled analysis showed no significant differences (OR [95% CI] = 0.76 [0.51–1.15], p = 0.20; Fig 4). The subgroup analyses by time and platform failed to show any significant differences.

### Other safety endpoints

Definite ST was available in 25 studies with 33325 patients. **(**S5 Table). Data on deaths were available in 37 studies with 37931 patients. **(**S6 Table). Cardiac death data were available in 32 studies with 35433 patients. **(**S7 Table). Data on myocardial infarction (MI) were available in 37 studies with 37923 patients. **(**S8 Table). BP-stainless DESs were associated with lower rates of long-term definite ST (OR [95% CI] = 0.71[0.53–0.96], p = 0.03) than other stents, with lower rates of very late definite ST (OR [95% CI] = 0.24[0.10–0.59], p = 0.002), long-term definite ST (OR [95% CI] = 0.63[0.43–0.93], p = 0.02) or long-term death (OR [95% CI] = 0.84[0.71–1.00], p = 0.05) than other stainless DESs and lower rates of short-term MI (OR [95% CI] = 0.60[0.38–0.95], p = 0.03) than BMSs. There were no other statistically significant differences in the safety endpoints of definite ST, death, cardiac death or MI.

### Efficacy endpoints:Target lesion revascularization (TLR)

The pooled analysis of TLR with BP-DESs and other stents included 32 studies with 31817 patients **(**S9 Table), and a significant difference was observed between these two groups (OR [95% CI] = 0.74[0.62–0.89], p = 0.001).

#### BP-stainless DES versus other stents

Twenty studies with 23069 patients compared BP-stainless stents to other stents. BP-stainless DESs had a lower risk of pooled TLR (OR [95% CI] = 0.73[0.59–0.91], p = 0.004), mid-time TLR (OR [95% CI] = 0.68[0.53–0.87], p = 0.003) and long-term TLR (OR [95% CI] = 0.72[0.55–0.94], p = 0.01) than other stents. The subgroup analysis revealed that BP-stainless stents were superior to other stainless DESs (OR [95% CI] = 0.73 [0.54–1.00], p = 0.05) and BMSs (OR [95% CI] = 0.33 [0.23–0.49], p<0.00001) in pooled TLR. The subgroup analysis also revealed a significant difference between BP-stainless DESs and other stainless DESs in long-term TLR (OR [95% CI] = 0.80[0.67–0.95], p = 0.01) and short-term TLR (OR [95% CI] = 0.58[0.35–0.97], p = 0.04) and between BP-stainless DESs and BMSs in mid-term TLR (OR [95% CI] = 0.29 [0.17–0.49], p <0.00001) and long-term TLR (OR [95% CI] = 0.32 [0.22–0.48], p <0.00001).

#### BP-alloy DES versus other stents

Twelve studies with 8748 patients compared BP-alloy DESs to other stents, and no significant differences were found in any of the variables examined.

### TVR

TVR data were extracted from 29 studies with 29761 patients **(**S10 Table) and were found to be significantly different between BP-DESs and other stents (OR [95% CI] = 0.76 [0.62–0.93], p = 0.008).

#### BP-stainless DES versus other stents

Nineteen studies with 21564 patients compared BP-stainless DESs with other stents. BP-stainless DESs were associated with a lower rate of pooled TVR (OR [95% CI] = 0.74 [0.58–0.96], p = 0.02), mid-term TVR (OR [95% CI] = 0.72 [0.55–0.94], p = 0.01) and long-term TVR (OR [95% CI] = 0.63 [0.44–0.91], p = 0.01) than other stents. The subgroup analysis also showed the superiority of BP-stainless DESs to BMSs in pooled TVR (OR [95% CI] = 0.34 [0.22–0.52], p <0.00001), mid-term TVR (OR [95% CI] = 0.29 [0.20–0.41], p <0.00001) and long-term TVR (OR [95% CI] = 0.34 [0.22–0.52], p <0.00001). No statistically significant differences were observed between BP-stainless DESs and other stents in any of the other variables analyzed.

#### BP-alloy DESs versus other stents

Ten studies with 8197 patients compared BP-alloy DESs to other stents. BP-alloy DESs were superior to other stents in short-term TVR (OR [95% CI] = 0.22[0.05–0.95], p = 0.04) and long-term TVR (OR [95% CI] = 0.31[0.14–0.68], p = 0.003). The subgroup analysis also demonstrated a statistically significant difference between BP-alloy DESs and BMSs (OR [95% CI] = 0.50 [0.28–0.92], p = 0.03) in pooled TVR. Regarding the other outcomes, no significant differences were observed between BP-alloy DESs and other stents.

### Additional analyses

A sensitivity analysis was conducted using the random-effects model. The results did not significantly change after the sequential removal of individual studies or after the removal of the article published in Chinese. Visual evaluation of the funnel plots showed no obvious publication bias **(**S2 Fig**).** After the predefined analysis, we performed a post-hoc analysis based on the duration of dual anti-platelet therapy in the pooled endpoints. After dividing the duration of DAPT into three subgroups of 3 months, 6 months and 12 months, we observed a significant difference between BP-DESs and other stents in 6 months TVR (OR [95% CI] = 0.55[0.33–0.92], p = 0.001) and in 12 months definite/probable ST (OR [95% CI] = 0.78[0.63–0.96], p = 0.001), TLR (OR [95% CI] = 0.75[0.59–0.95], p = 0.001).

## Discussion

In comparison to prior meta-analyses, our analysis included the following novel features: 1. We conducted the first evaluation the efficacy and safety of BP-DESs with different platforms (BP-stainless and BP-alloys). 2. BP-DESs were found to be superior to other stents in definite/probable ST, TLR, and TVR in the pooled analysis. 3. BP-stainless DESs had lower rates of pooled, long-term and very late definite/probable ST, pooled, mid-term and long-term TLR, TVR than other stents. Similar results were observed when the control groups used were other stainless DESs and BMSs. 4. BP-stainless DESs were superior to other alloy DESs in late definite/probable ST. 5. BP-alloy DESs were superior to other stents in the short-term and long-term TVR and were superior to BMSs in pooled TVR.

Whether the stent platform affects the clinical safety and efficacy of BP-DESs is unclear. A pre-clinical study comparing[17] stents with identical structures, platforms and release kinetics but different coating drugs—everolimus (EES), sirolimus (SES) or zotarolimus (ZES)—has shown similar outcomes, thus suggesting that stent backbones rather than the “-limus” drugs are responsible for the vessel response. Currently, primarily two metal materials are used as stent struts: stainless steel and alloys. Stainless steel is a biologically inert metal and was the first material used in the design of coronary stents[18]. In the era of BMSs, evidence suggested that a low stent strut thickness improved the safety of the stent[13]. Therefore, metallic alloys, such as cobalt–chromium (CoCr), were developed with increased levels of radiopacity and strength, allowing struts to be much thinner[18]. However, alloys also have disadvantages[19]. For example, CoCr is associated with higher elastic properties and greater stent recoil, which might be disadvantageous clinically. Platinum-chromium is a new alloy used in the design of stent platforms that are intended to have improved biocompatibility and strength, but evidence regarding the clinical safety and efficacy of platinum-chromium in BP-DESs is minimal[20]. In recent years, many RCTs have indirectly compared BP-DESs with different platforms to other stents, but no meta-analysis has focused on platform classification. With 34,850 randomized individuals, our study has sufficient power to determine whether platform material affects the clinical endpoints of BP-DESs implantation.

The superiority of BP-stainless DESs over other stainless DESs and BMSs is mainly shown by the results of the pooled analysis of definite/probable ST, TLR and TVR. Previous studies[3,4] have suggested that the polymer remnants of first-generation DESs trigger a chronic inflammatory response, which causes delayed healing of the vessel wall and ST. Some studies[11,21,22] have also shown that BP-BESs are associated with significantly reduced risks of TLR and TVR compared with first-generation DESs [especially paclitaxel-eluting stents (PES, Taxus)] and BMSs. As in the present study, the control stainless stents were mainly first-generation DESs, such as sirolimus-eluting stents (Cypher, SES) and PESs. Therefore, we speculated that the superiority of BP-DESs might be due to the poor efficiency profile of the control stents rather than to a particular advantage of the BP-DESs. To confirm this hypothesis, large-scale randomized studies comparing BP-stainless DESs to other new-generation stainless stents are necessary.

In the comparison of BP-stainless DESs with other alloy DESs, only the rate of the late definite/probable ST event was significantly lower. Previous random clinical trials, such as COMPARE II[23] and NEXT[24], have shown that the outcomes of BP-stainless DES implantation are not inferior to those of other alloy DESs. Therefore, BP-stainless DESs do not appear to be inferior to alloy DESs. However, the results of recent comprehensive meta-analyses have not supported this finding; those meta-analyses have found that stainless BP-BESs are inferior in ST to one particular alloy DES (CoCr-EES)[11, 21, 22]. Possible reasons for these contradictory findings are as follows: 1. The network meta-analysis had its own limitations, and the indirect evidence of those analyses might not support real world practice; 2. In our meta-analysis, we evaluated only the overall effect of different BP-DESs with the same platform material. Furthermore, in the present study, the observed significant endpoint was reported by only four studies, and one study showing significant benefits of BP-stainless DESs included only ST-segment elevation myocardial infarction (STEMI) patients. Therefore, the results of this meta-analysis must be considered with caution, and whether BP-stainless DESs and other alloy DESs have different efficacies remains to be verified in future rigorously designed random clinical trials.

Interestingly, BP-alloy DESs, compared with other stents, showed few significant results. This finding might seem contrary to the perception that metallic alloys are more suitable stent platforms because they allow for thinner struts and better stent flexibility[10,17,19], so we will elaborate on this observation finding in two aspects. First, BP-alloy DESs were not superior to other stainless DESs. The possible explanations for this finding are as follows: (i). Animal experiments[25] comparing stainless steel and CoCr stents in a swine model showed that despite the lower strut thickness, CoCr stents were not superior to stainless steel stents in neointimal hyperplasia inhibition. It appears that improvement of the physical properties of the platform might not translate into clinical safety and efficacy benefits, especially in the era of BP-DESs. (ii). Only three trials compared BP-alloy DESs with other stainless DESs, and future RCTs should focus on comparing these types of DESs to provide more valuable data. Second, BP-alloy DESs were superior to other alloy DESs in only certain aspects. This result may be explained as follows: (i). The controlled alloy DESs all belong to the second-generation DESs, whose safeties and efficacies have been proven to be better than those of first-generation DESs[17,22]. Therefore, the improvement of the control group compared with first-generation DESs might be partially responsible for the non-superior results. (ii). The types of BP-alloy DESs examined presented more heterogeneity among articles than BP-stainless DESs. For example, in the INSPIRON-I[26] study, the stent used was Inspiron BP-DES, whereas in the BIOSCIENCE[13] study, the stent used was Orsiro BP-DES. To conclude, based on the current analysis, we believe that BP-alloy DESs are not inferior to other stents. However, future large-scale, well-design RCTs are needed to confirm this conclusion.

### Limitations

The limitations of our meta-analysis are as follows: (i). As with any meta-analysis, the limitations of each included trial influences the results. (ii). BMS was included and was regarded as a single comparator; this might have influenced the results of some endpoints. (iii). We included many studies in which first-generation DESs served as the control stents; this may have affected the overall analysis. (iv). We included only articles published in English or Chinese; this may have increased the selection bias. (v). Our meta-analysis was aimed at examining the effect of the stent platform type. However, for BP-DESs, both the polymer materials and coating drugs affect clinical outcomes; therefore, large-scaled RCTs comparing BP-DESs with identical polymers and drugs but different platforms are needed to further evaluate the effect of struts on outcomes. (vi). The duration of DAPT ranged from 3 months to ≥12 months, which could be a possible confounding factor. (vii): Relatively fewer articles compared BP-alloy DESs with other stents than compared BP-stainless DESs with other stents. Furthermore, the types of BP-alloy DESs used were more heterogeneous than the types of BP-stainless DESs used.

## Conclusion

The present meta-analysis of 40 trials (49 studies) comprising 34,850 randomized patients showed that BP-stainless DESs are associated with superior pooled, long-term and very late definite/probable ST compared with other stents (mainly stainless DESs); superior long-term definite ST compared with other stents (mainly stainless DESs); and superior pooled, mid-term and long-term TVR and TLR compared with other stents (mainly stainless DESs and BMSs). BP-alloy DESs were relatively less beneficial than other stents in all other variables examined. These results suggest that BP-stainless DESs may offer more benefits than BP-alloy DESs in the era of BP-DESs.

## Supporting Information

S1 PRISMA Checklist PRISMA checklist. (DOC)

S1 Fig Risk of bias graph. (TIF)

S2 Fig Visual funnel plots of pooled definite/probable ST of BP-DESs and other stents. (TIF)

S1 Table Search strategy on PubMed. (DOC)

S2 Table Characteristics of included trials. (DOC)

S3 Table Characteristics of included patients. (DOC)

S4 Table Definite/probable stent thrombosis. (DOC)

S5 Table Definite stent thrombosis. (DOCX)

S6 Table Death. (DOC)

S7 Table Cardiac death. (DOC)

S8 Table Myocardial infarction. (DOC)

S9 Table Target lesion revascularization. (DOC)

S10 Table Target vessel revascularization. (DOC)
