# Supplementary figures and images for: Can Platforms Affect the Safety and Efficacy of Drug-Eluting Stents in the Era of Biodegradable Polymers?: A Meta-Analysis of 34,850 Randomized Individuals
Source: PLoS One. 2016 Mar 31;11(3):e0151259. doi: 10.1371/journal.pone.0151259 (PMC4816558; doi:10.1371/journal.pone.0151259)

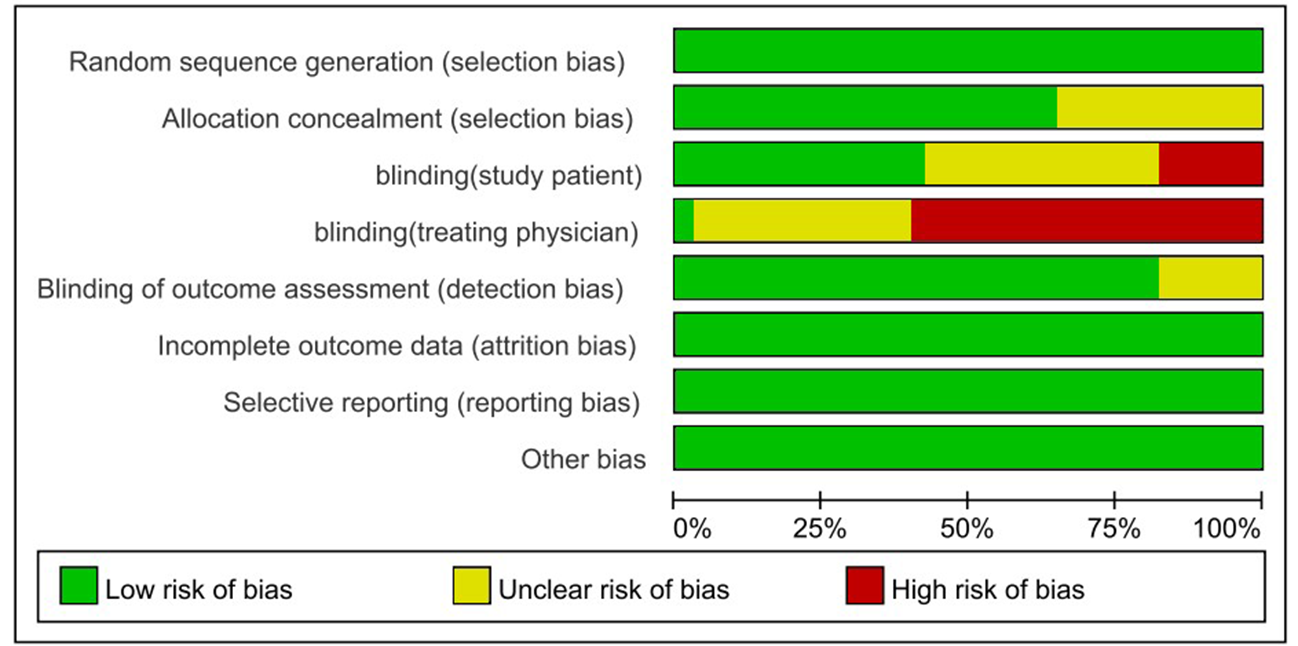

Supplement: S1 Fig — (TIF) [file pone.0151259.s002.tif]

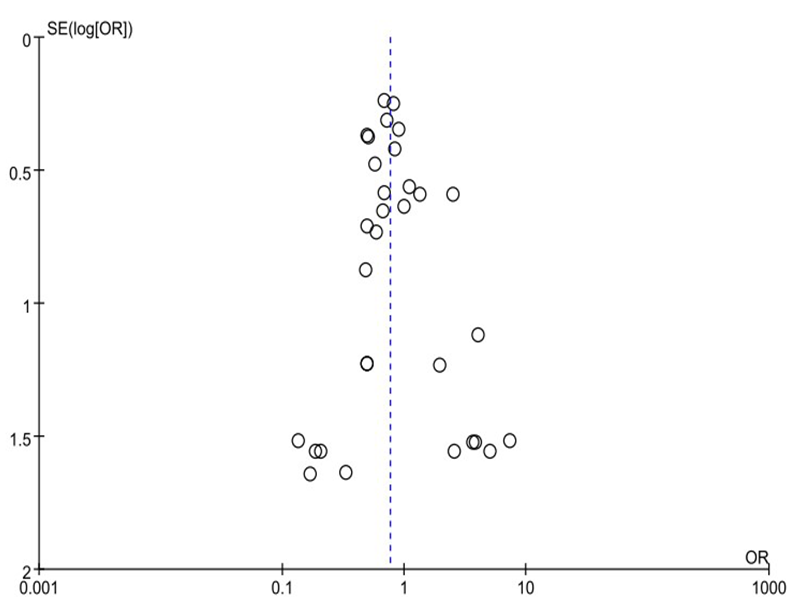

Supplement: S2 Fig — (TIF) [file pone.0151259.s003.tif]
