# Supplementary material for: Can Platforms Affect the Safety and Efficacy of Drug-Eluting Stents in the Era of Biodegradable Polymers?: A Meta-Analysis of 34,850 Randomized Individuals
Source: PLoS One. 2016 Mar 31;11(3):e0151259. doi: 10.1371/journal.pone.0151259 (PMC4816558; doi:10.1371/journal.pone.0151259)
Supplement: S1 Table — (DOC) [file pone.0151259.s004.doc]

**S1 Table. Search strategy on PubMed**

| **Pubmed** | |
| --- | --- |
| #1 | "Percutaneous Coronary Intervention"[Mesh] |
| #2 | "Coronary Disease"[Mesh] |
| #3 | "PCI" |
| #4 | "CAD" |
| #5 | (#1) OR (#2) OR (#3) OR (#4) |
| #6 | "biodegradable"[tiab] |
| #7 | "degradable"[tiab] |
| #8 | "bioabsorbable"[tiab] |
| #9 | "absorbable"[tiab] |
| #10 | "absorptive"[tiab] |
| #11 | "dissolvable"[tiab] |
| #12 | (#6) OR (#7) OR (#8) OR (#9) OR (#10) OR (#11) |
| #13 | "Polymers"[Mesh] OR "Polymer"[tiab] OR "coating"[tiab] |
| #14 | (#12) AND (#13) |
| #15 | "BioMatrix" OR "NOBORI" OR "Axxess" OR "Supralimus" OR "Infinnium" OR "BioMime" OR "Orsiro" OR "DESyne" OR "SYNERGY" OR "MiStent" OR "Excel" OR "Firehawk" OR "NOYA" OR "Inspiron" OR "Tivoli" OR "BuMA" OR "Svelte" OR "Custom" OR "NEVO" OR "Elixir" OR "JACTAX" OR "CORACTO" |
| #16 | (#14)) OR (#15) |
| #17 | "randomized controlled trial"[pt] OR "controlled clinical trial"[pt] OR "randomized"[tiab] OR "placebo"[tiab] OR "drug therapy"[sh] OR "randomly"[tiab] OR "trial"[tiab] OR "groups"[tiab] |
| #18 | "animals"[Mesh] NOT "humans"[Mesh] |
| #19 | # (17) NOT (#18) |
| #20 | # (5) AND # (16) AND # (19) |
