# Supplementary material for: Can Platforms Affect the Safety and Efficacy of Drug-Eluting Stents in the Era of Biodegradable Polymers?: A Meta-Analysis of 34,850 Randomized Individuals
Source: PLoS One. 2016 Mar 31;11(3):e0151259. doi: 10.1371/journal.pone.0151259 (PMC4816558; doi:10.1371/journal.pone.0151259)
Supplement: S2 Table — (DOC) [file pone.0151259.s005.doc]

**S2 Table. Characteristics of included trials**

| Trials | Stent comprision(brand)  (sample size) | Primary endpoint | design | Major inclusion criteria | Maximal lengh of follow up | DAPT duration | arms | language |
| --- | --- | --- | --- | --- | --- | --- | --- | --- |
| Published in 2004 | | | | | | | | |
| FUTUREⅠ | BP-stainless DES (27) | Composite of death CABG MI and TLR at 30 days | single-center | All-comer design | 1 year | ≥6 months | 2 | English |
|  | BMS(15) |  |  |  |  |  |  |  |
| Published in 2005 | | | | | | | | |
| STEALTH | BP-stainless DES (BioMatrix) (80) | In-stent LLL at 6 months | Multicenter, superiority | Stable or unstable angina | 6 months | ≥3 months | 2 | English |
|  | BMS(40) |  |  |  |  |  |  |  |
| Published in 2007 | | | | | | | | |
| Nobori 1 | BP-stainless DES (Nobori) (85) | In-stent LL at 9 months | Multicenter non-inferiority | Stable or unstable angina | 9 months | ≥6 months | 2 | English |
|  | DP-stainless DES (Taxus) (35) |  |  |  |  |  |  |  |
| Published in 2008 | | | | | | | | |
| ISAR-TEST-3, | BP-stainless DES (202) | In-stent LLL | Multicenter non-inferiority | Stable or unstable angina | 2 years | ≥1 year | 3 | English |
|  | DP-stainless DES (Cypher)(202) |  |  |  |  |  |  |  |
|  | PF-stainless DES(201) |  |  |  |  |  |  |  |
| LEADERS,, | BP-stainless DES (BioMatrix) (857) | Composite of cardiac death, MI, TVR at 9 months | Multicenter, non-inferiority | Stable angina, ACS | 5 years | ≥1 year | 2 | English |
|  | DP-stainless DES (Cypher SELECT) (850) |  |  |  |  |  |  |  |
| COSTAR II | BP-alloy DES (CoStar) (989) | Composite of adjudicated death, MI, TVR at 8 months | Multicenter, non-inferiority | All-comer design | 8 months | ≥6 months | 2 | English |
|  | DP-stainless DES (Taxus) (686) |  |  |  |  |  |  |  |
| Published in 2009 | | | | | | | | |
| ISAR-TEST-4, | BP- stainless DES (Yukon) (1299) | composite of cardiac death, MI, TLR at 12 months | Multicenter non-inferiority | Stable or unstable angina | 5 years | ≥6 months | 3 | English |
|  | DP- stainless DES (Cypher) (652) |  |  |  |  |  |  |  |
|  | DP-alloy DES (Xience) (652) |  |  |  |  |  |  |  |
| PAINT, | BP- Stainless DES (Infinnium) (111) | in-stent LLL at 9 months | Multicenter | All-comer design | 5 years | ≥1 year | 3 | English |
|  | BP-Stainless DES (Supralimus) (106) |  |  |  |  |  |  |  |
|  | BMS (57) |  |  |  |  |  |  |  |
| NOBORI 1—Phase 2 | BP-stainless DES (Nobori)(153) | In-stent LL at 9 months | Multicenter non-inferiority | Stable or unstable angina | 9 months | ≥6 months | 2 | English |
|  | DP- stainless DES (Taxus Liberte) (90) |  |  |  |  |  |  |  |
| Published in 2010 | | | | | | | | |
| Li Q | BP-stainless DES (Exce1) (115) | composite of death, MI, TLR or TVR at 12 months | single-center | STEMI | 1 year | ≥1 year | 2 | Chinese |
|  | DP-stainless DES (Cypher Select) (113) |  |  |  |  |  |  |  |
| EXCELLA II | BP-alloy DES（Elixir Medical）(139) | in-stent LLL at 9-months | Multicenter non-inferiority | Stable or unstable angina | 9 months | ≥1 year | 2 | English |
|  | DP-alloy DES (Endeavor) (71) |  |  |  |  |  |  |  |
| NEVO RES-I, | BP-alloy DES (NEVO) (202) | in-stent LLL at 6 months | Multicenter non-inferiority or superiority | Stable or unstable angina | 2 years | ≥6 months | 2 | English |
|  | DP-stianless DES (TAXUS) (192) |  |  |  |  |  |  |  |
| CORACTO | BP-stainless DES (CORACTO) (48) | in-segment LLL and restenosis after six months | unclear | CTO | 2 year | ≥6 months | 2 | English |
|  | BMS (47) |  |  |  |  |  |  |  |
| Published in 2011 | | | | | | | | |
|  | | | | | | | | |
| EUROSTAR-II | BP-alloy DES (CoStar) (152) | in-segment binary restenosis rate at 8 months | Multicenter superiority | Stable or unstable angina | 8 months | ≥6 months | 2 | English |
|  | BMS（151） |  |  |  |  |  |  |  |
| Separham A | BP-stainless DES (BioMatrix) (100) | composite of cardiac death, MI and TVR at 12 months | single-center | Stable angina, ACS | 1 year | ≥1 year | 2 | English |
|  | DP-alloy DES (Xience V) (100) |  |  |  |  |  |  |  |
| Published in 2012 | | | | | | | | |
| Zhang L | BP-stainless DES (EXCEL) (85) | composite of cardiac death, MI, TLR | single-center noninferiority or equivalence | Stable angina, ACS | 1 year | ≥1 year | 2 | English |
|  | PF-stainless DES (YINYI) (82) |  |  |  |  |  |  |  |
| NOYA I | BP-alloy DES (NOYA) (150) | in-stent LLL at 9 months | Multicenter, non-inferiority | All-comer design | 2 years | ≥1 year | 2 | English |
|  | DP-alloy DES (FIREBIRD2) (150) |  |  |  |  |  |  |  |
| EVOLVE, | BP-alloy DES (SYNERGY) (94) | composite of cardiac death, MI, TLR at 30 days | Multicenter non-inferiority | Stable or unstable angina | 2 years | ≥6 months | 3 | English |
|  | BP-alloy DES (SYNERGY half dose) (99) |  |  |  |  |  |  |  |
|  | DP-alloy DES (PROMUS Element) (98) |  |  |  |  |  |  |  |
| COMFORTABLE AMI, | BP-stainless DES (BioMatrix）(575) | composite of cardiac death, MI, and TLR at 1 year | Multicenter superiority | AMI | 2 years | ≥1 year | 2 | English |
|  | BMS (582) |  |  |  |  |  |  |  |
| Kadota K | BP-stainless DES (Nobori) (198) | composite of cardiac death, MI, TVR at 9 months | Multicenter, non-inferiority | Stable or unstable angina | 9 months | ≥3 months | 2 | English |
|  | DP- stainless DES (Cypher) (137) |  |  |  |  |  |  |  |
| Published in 2013 | | | | | | | | |
| TARGET I | BP-alloy DES (FIREHAWK) (227) | in-stent LLL at 9 months | Multicenter, non-inferiority | single de novo native coronary artery lesion | 1 year | ≥1 year | 2 | English |
|  | DP-alloy DES (XIENCE V) (231) |  |  |  |  |  |  |  |
| ZHANG Y | BP-stainless DES (Excel) (341) | composite of cardiac death, MI, TLR at 2 years | Multicenter non-inferiority | Stable or ACS | 2 years | ≥1 year | 3 | English |
|  | PF- stainless DES (Yinyi) (327) |  |  |  |  |  |  |  |
|  | DP- stainless DES (Partner) (321) |  |  |  |  |  |  |  |
| DKPLUS-Wave 1 | BP-stainless DES (Excel) (173) | TVR at 12 months | Multicenter | Stable or unstable angina | 1 year | ≥6 months | 2 | English |
|  | PF-stainless DES (Real Dual) (173) |  |  |  |  |  |  |  |
| COMPARE II, | BP-stainless DES（Nobori）(1795) | Composite cardiac death, MI, TVR at 12 months | Multicenter, non-inferiority | All-comer design | 3 year | ≥1 year | 2 | English |
|  | DP-alloy DES（Xience V, Prime or Promus）(912) |  |  |  |  |  |  |  |
| NEXT | BP-stianless DES（Nobori）(1617) | TLR at 1 year | Multicenter, non-inferiority | All-comer design | 1 year | unclear | 2 | English |
|  | DP-alloy DES (Xience V or Promus) (1618) |  |  |  |  |  |  |  |
| SORT OUT V | BP-stainless DES (Nobori) (1229) | composite of cardiac death, MI, definite ST, TVR at 9 months | Multicenter non-inferiority | all-comer design | 1 year | ≥1 year | 2 | English |
|  | DP- stainless DES （Cypher Select Plus）(1239) |  |  |  |  |  |  |  |
| Li Q | BP-stainless DES (Excel) (164) | composite of cardiac death, recurrent infarction, and TVR at 3 years | single-center | STEMI | 3 years | ≥1 year | 2 | English |
|  | DP-stainless DES (Cypher Select) (168) |  |  |  |  |  |  |  |
| Published in 2014 | | | | | | | | |
| INSPIRON-I | BP-alloy DES (Inspiron) (38) | in-segment LLL at 6 months | Multicenter | Stable or unstable angina | 2 year | NA | 2 | English |
|  | BMS (19) |  |  |  |  |  |  |  |
| BIOSCIENCE | BP-alloy DES (Orsiro) (1063) | composite of cardiac death, MI, TLR within 12 months | Multicenter, non-inferiority | All-comer design | 1 year | ≥1 year | 2 | English |
|  | DP-alloy DES (Xience Prime) (1056) |  |  |  |  |  |  |  |
| HOPE | BP-alloy DES (HELIOS) (142) | in-stent LLL at 9 months | Multicenter non-inferiority | Stable or unstable angina | 3 year | ≥1 year | 2 | English |
|  | DP-stainless DES (PARTNER) (145) |  |  |  |  |  |  |  |
| I-LOVE-IT 2 | BP-alloy DES (Tivoli) (1829) | composite of cardiac death, MI，TLR at 12 months | Multicenter, non-inferiority | All-comer design | 1 year | ≥6 months | 2 | English |
|  | DP-alloy DES (Firebird 2) (908) |  |  |  |  |  |  |  |
| LONG-DES | BP-stainless DES (Nobori) (245) | in-segment LLL at 9 months | Multicenter | Stable or unstable angina or NSTEMI | 1 year | ≥1 year | 2 | English |
|  | DP-alloy DES (Promus Element）(255) |  |  |  |  |  |  |  |
| RESOLVE | BP-stainless DES (EXCEL) (596) | Composite of death, MI, TLR at 1 year | Multicenter noninferiority | STEMI | 1 year | ≥1 year | 2 | English |
|  | DP-alloy DES (Firebird or Firebird II) (596) |  |  |  |  |  |  |  |
| CENTURY II | BP-alloy DES (Ultimaster) (711) | composite of cardiac death, MI, TLR at 9 months | Multicenter, non-inferiority | All-comer design | 9 months | ≥6 months | 2 | English |
|  | DP-alloy DES (Xinece) (716) |  |  |  |  |  |  |  |
| Published in 2015 | | | | | | | | |
| DESSOLVE II | BP-alloy DES (APSES) (123) | in-stent LLL at 9 months | Multicentre superiority | Stable or unstable angina | 9 months | ≥1 year | 2 | English |
|  | DP-alloy DES (Endeavor) (61) |  |  |  |  |  |  |  |
| SORT OUT VI | BP-stainless DES (BioMatrix Flex) (1497) | combination of cardiac death, MI, and TLR within 12 months | Multicenter, non-inferiority | Stable angina or ACS | 1 year | ≥1 year | 2 | English |
|  | DP-alloy DES (Resolute Integrity) (1502) |  |  |  |  |  |  |  |
| EVERBIO II | BP-stainless DES (Biomatrix Flex) (80) | in-stent LLL at 9 months | single-center, superiority | Stable angina or ACS | 9 months | ≥12 months | 2 | English |
|  | DP-alloy DES (Promus Element) (80) |  |  |  |  |  |  |  |
| BASKET-PROVE II | BP-stainless DES (Nobori) (765) | combination of cardiac death, MI, TVR within 2 years | Multicenter, non-inferiority | Stable or ACS | 2 years | ≥1 year | 3 | English |
|  | DP-alloy DES (Xience prime) (765) |  |  |  |  |  |  |  |
|  | BMS (761) |  |  |  |  |  |  |  |
| Shen L | BP-stainless DES (105) | combination of cardiac death, MI, TVR at 2 years | Multicenter | Stable or unstable angina | 2 year | ≥6 months | 2 | English |
|  | DP-alloy DES (FIREBIRD) (107) |  |  |  |  |  |  |  |
| BIOFLOW-II | BP-alloy DES (Orsiro) (298) | in-stent LLL at 9 months | Multicenter noninferiority | Stable or unstable angina | 1 year | ≥6 months | 2 | English |
|  | DP-alloy DES (Xience Prime) (154) |  |  |  |  |  |  |  |

BP indicates biodegradable polymer; DP indicates durable polymer; PF indicates polymer free; DES indicates drug-eluting stent; BMS indicates bare metal stent; ST indicates stent thrombosis; MI indicates myocardial infarction; TLR indicates target lesion revascularization; TVR indicates target vessel revascularization; LLL indicates late lumen loss; ACS indicates acute coronary syndrome; STEMI indicates ST-segment elevation myocardial infarction; AMI indicates acute myocardial infarction; CTO indicates chronic total coronary occlusions; CABG indicates coronary artery bypass grafting; DAPT indicates dual anti-platelet therapy; NA indicates not available.

**Reference**

1. Grube E, Sonoda S, Ikeno F, Honda Y, Kar S, Chan C, et al. Six- and twelve-month results from first human experience using everolimus-eluting stents with bioabsorbable polymer. Circulation. 2004;109(18):2168-71. doi: 10.1161/01.CIR.0000128850.84227.FD. PubMed PMID: 15123533.

2. Grube E, Hauptmann KE, Buellesfeld L, Lim V, Abizaid A. Six-month results of a randomized study to evaluate safety and efficacy of a Biolimus A9 eluting stent with a biodegradable polymer coating. EuroIntervention : journal of EuroPCR in collaboration with the Working Group on Interventional Cardiology of the European Society of Cardiology. 2005;1(1):53-7. Epub 2005/05/01. PubMed PMID: 19758877.

3. Chevalier B, Serruys PW, Silber S, Garcia E, Suryapranata H, Hauptmann K, et al. Randomised comparison of Nobori, biolimus A9-eluting coronary stent with a Taxus(R), paclitaxel-eluting coronary stent in patients with stenosis in native coronary arteries: the Nobori 1 trial. EuroIntervention : journal of EuroPCR in collaboration with the Working Group on Interventional Cardiology of the European Society of Cardiology. 2007;2(4):426-34. Epub 2007/02/01. PubMed PMID: 19755281.

4. Mehilli J, Byrne RA, Wieczorek A, Iijima R, Schulz S, Bruskina O, et al. Randomized trial of three rapamycin-eluting stents with different coating strategies for the reduction of coronary restenosis. European heart journal. 2008;29(16):1975-82. Epub 2008/06/14. doi: 10.1093/eurheartj/ehn253. PubMed PMID: 18550554.

5. Byrne RA, Kufner S, Tiroch K, Massberg S, Laugwitz KL, Birkmeier A, et al. Randomised trial of three rapamycin-eluting stents with different coating strategies for the reduction of coronary restenosis: 2-year follow-up results. Heart. 2009;95(18):1489-94. doi: 10.1136/hrt.2009.172379. PubMed PMID: 19592388.

6. Windecker S, Serruys PW, Wandel S, Buszman P, Trznadel S, Linke A, et al. Biolimus-eluting stent with biodegradable polymer versus sirolimus-eluting stent with durable polymer for coronary revascularisation (LEADERS): a randomised non-inferiority trial. The Lancet. 2008;372(9644):1163-73. doi: 10.1016/s0140-6736(08)61244-1.

7. Garg S, Sarno G, Serruys PW, de Vries T, Buszman P, Linke A, et al. The twelve-month outcomes of a biolimus eluting stent with a biodegradable polymer compared with a sirolimus eluting stent with a durable polymer. EuroIntervention : journal of EuroPCR in collaboration with the Working Group on Interventional Cardiology of the European Society of Cardiology. 2010;6(2):233-9. Epub 2010/06/22. doi: 10.4244/. PubMed PMID: 20562074.

8. Serruys PW, Farooq V, Kalesan B, de Vries T, Buszman P, Linke A, et al. Improved safety and reduction in stent thrombosis associated with biodegradable polymer-based biolimus-eluting stents versus durable polymer-based sirolimus-eluting stents in patients with coronary artery disease: final 5-year report of the LEADERS (Limus Eluted From A Durable Versus ERodable Stent Coating) randomized, noninferiority trial. JACC Cardiovascular interventions. 2013;6(8):777-89. doi: 10.1016/j.jcin.2013.04.011. PubMed PMID: 23968698.

9. Krucoff MW, Kereiakes DJ, Petersen JL, Mehran R, Hasselblad V, Lansky AJ, et al. A novel bioresorbable polymer paclitaxel-eluting stent for the treatment of single and multivessel coronary disease: primary results of the COSTAR (Cobalt Chromium Stent With Antiproliferative for Restenosis) II study. Journal of the American College of Cardiology. 2008;51(16):1543-52. doi: 10.1016/j.jacc.2008.01.020. PubMed PMID: 18420096.

10. Byrne RA, Kastrati A, Kufner S, Massberg S, Birkmeier KA, Laugwitz KL, et al. Randomized, non-inferiority trial of three limus agent-eluting stents with different polymer coatings: the Intracoronary Stenting and Angiographic Results: Test Efficacy of 3 Limus-Eluting Stents (ISAR-TEST-4) Trial. European heart journal. 2009;30(20):2441-9. Epub 2009/09/02. doi: 10.1093/eurheartj/ehp352. PubMed PMID: 19720642.

11. Kufner S, Byrne RA, Valeskini M, Schulz S, Ibrahim T, Hoppmann P, et al. Five-year outcomes from a trial of three limus-eluting stents with different polymer coatings in patients with coronary artery disease: final results from the ISAR-TEST 4 randomised trial. EuroIntervention : journal of EuroPCR in collaboration with the Working Group on Interventional Cardiology of the European Society of Cardiology. 2014. doi: 10.4244/EIJY14M11_02. PubMed PMID: 25405657.

12. Lemos PA, Moulin B, Perin MA, Oliveira LA, Arruda JA, Lima VC, et al. Randomized evaluation of two drug-eluting stents with identical metallic platform and biodegradable polymer but different agents (paclitaxel or sirolimus) compared against bare stents: 1-year results of the PAINT trial. Catheterization and cardiovascular interventions : official journal of the Society for Cardiac Angiography & Interventions. 2009;74(5):665-73. Epub 2009/08/12. doi: 10.1002/ccd.22166. PubMed PMID: 19670303.

13. Marchini JF, Gomes WF, Moulin B, Perin MA, Oliveira LA, Arruda JA, et al. Very late outcomes of drug-eluting stents coated with biodegradable polymers: insights from the 5-year follow-up of the randomized PAINT trial. Cardiovascular diagnosis and therapy. 2014;4(6):480-6. doi: 10.3978/j.issn.2223-3652.2014.12.05. PubMed PMID: 25610805; PubMed Central PMCID: PMC4278035.

14. Chevalier B, Silber S, Park SJ, Garcia E, Schuler G, Suryapranata H, et al. Randomized comparison of the Nobori Biolimus A9-eluting coronary stent with the Taxus Liberte paclitaxel-eluting coronary stent in patients with stenosis in native coronary arteries: the NOBORI 1 trial--Phase 2. Circulation Cardiovascular interventions. 2009;2(3):188-95. Epub 2009/12/25. doi: 10.1161/circinterventions.108.823443. PubMed PMID: 20031715.

15. Li Q, Wang LF, Yang XC, Ge YG, Wang HS, Li WM, et al. [Efficacy comparison of primary percutaneous coronary intervention with biodegradable polymer- and durable polymer-based sirolimus-eluting stents for patients with acute myocardial infarction]. Zhonghua xin xue guan bing za zhi. 2010;38(10):886-90.

16. Serruys PW, Garg S, Abizaid A, Ormiston J, Windecker S, Verheye S, et al. A randomised comparison of novolimus-eluting and zotarolimus-eluting coronary stents: 9-Month follow-up results of the EXCELLA II study. EuroIntervention : journal of EuroPCR in collaboration with the Working Group on Interventional Cardiology of the European Society of Cardiology. 2010;6(2):195-205.

17. Ormiston JA, Abizaid A, Spertus J, Fajadet J, Mauri L, Schofer J, et al. Six-month results of the NEVO res-elution I (NEVO RES-I) Trial : A randomized, multicenter comparison of the nevo sirolimus-eluting coronary stent with the taxus liberte paclitaxel-eluting stent in de novo native coronary artery lesions. Circulation: Cardiovascular Interventions. 2010;3(6):556-64.

18. Abizaid A, Ormiston JA, Fajadet J, Mauri L, Schofer J, Verheye S, et al. Two-year follow-up of the NEVO ResElution-I(NEVO RES-I) trial: A randomised, multicentre comparison of the NEVO sirolimus-eluting coronary stent with the TAXUS Liberte paclitaxel-eluting stent in de novo native coronary artery lesions. EuroIntervention : journal of EuroPCR in collaboration with the Working Group on Interventional Cardiology of the European Society of Cardiology. 2013;9(6):721-9.

19. Reifart N, Hauptmann KE, Rabe A, Enayat D, Giokoglu K. Short and long term comparison (24 months) of an alternative sirolimus-coated stent with bioabsorbable polymer and a bare metal stent of similar design in chronic coronary occlusions: the CORACTO trial. EuroIntervention : journal of EuroPCR in collaboration with the Working Group on Interventional Cardiology of the European Society of Cardiology. 2010;6(3):356-60. Epub 2010/10/05. doi: 10.4244/eijv6i3a59. PubMed PMID: 20884414.

20. Silber S, Gutierrez-Chico JL, Behrens S, Witzenbichler B, Wiemer M, Hoffmann S, et al. Effect of paclitaxel elution from reservoirs with bioabsorbable polymer compared to a bare metal stent for the elective percutaneous treatment of de novo coronary stenosis: The EUROSTAR-II randomised clinical trial. EuroIntervention : journal of EuroPCR in collaboration with the Working Group on Interventional Cardiology of the European Society of Cardiology. 2011;7(1):64-73.

21. Separham A, Sohrabi B, Aslanabadi N, Ghaffari S. The twelve-month outcome of biolimus eluting stent with biodegradable polymer compared with an everolimus eluting stent with durable polymer. Journal of cardiovascular and thoracic research. 2011;3(4):113-6. Epub 2011/01/01. doi: 10.5681/jcvtr.2011.025. PubMed PMID: 24250967; PubMed Central PMCID: PMCPmc3825338.

22. Zhang L, Yuan J, Liu G, Zhong JP, Yin YH, She Q, et al. One-year clinical outcome of a randomized trial of polymer-free paclitaxel-eluting stents versus biodegradable polymer-based rapamycin-eluting stents in patients with coronary heart disease. Journal of interventional cardiology. 2012;25(6):604-10. Epub 2012/03/06. doi: 10.1111/j.1540-8183.2012.00722.x. PubMed PMID: 22384973.

23. Xu B, Dou K, Yang Y, Lv S, Wang L, Wang H, et al. Nine-month angiographic and 2-year clinical follow-up of the NOYA biodegradable polymer sirolimus-eluting stent in the treatment of patients with de novo native coronary artery lesions: The NOYA I trial. EuroIntervention : journal of EuroPCR in collaboration with the Working Group on Interventional Cardiology of the European Society of Cardiology. 2012;8(7):796-802.

24. Meredith IT, Verheye S, Dubois CL, Dens J, Fajadet J, Carrie D, et al. Primary endpoint results of the EVOLVE trial: A randomized evaluation of a novel bioabsorbable polymer-coated, everolimus-eluting coronary stent. Journal of the American College of Cardiology. 2012;59(15):1362-70.

25. Meredith IT, Verheye S, Weissman NJ, Barragan P, Scott D, Chavarri MV, et al. Six-month IVUS and two-year clinical outcomes in the EVOLVE FHU trial: A randomised evaluation of a novel bioabsorbable polymer-coated, everolimus-eluting stent. EuroIntervention : journal of EuroPCR in collaboration with the Working Group on Interventional Cardiology of the European Society of Cardiology. 2013;9(3):308-15.

26. Raber L, Kelbaek H, Ostoijc M, Baumbach A, Heg D, Tuller D, et al. Effect of biolimus-eluting stents with biodegradable polymer vs bare-metal stents on cardiovascular events among patients with acute myocardial infarction: The comfortable AMI randomized trial. JAMA - Journal of the American Medical Association. 2012;308(8):777-87.

27. Raber L, Kelbaek H, Taniwaki M, Ostojic M, Heg D, Baumbach A, et al. Biolimus-eluting stents with biodegradable polymer versus bare-metal stents in acute myocardial infarction: two-year clinical results of the COMFORTABLE AMI trial. Circulation Cardiovascular interventions. 2014;7(3):355-64. Epub 2014/05/23. doi: 10.1161/circinterventions.113.001440. PubMed PMID: 24847017.

28. Kadota K, Muramatsu T, Iwabuchi M, Saito S, Hayashi Y, Ikari Y, et al. Randomized comparison of the nobori biolimus A9-eluting stent with the sirolimus-eluting stent in patients with stenosis in native coronary arteries. Catheterization and Cardiovascular Interventions. 2012;80(5):789-96.

29. Gao RL, Xu B, Lansky AJ, Yang YJ, Ma CS, Han YL, et al. A randomised comparison of a novel abluminal groove-filled biodegradable polymer sirolimus-eluting stent with a durable polymer everolimus-eluting stent: Clinical and angiographic follow-up of the TARGET I trial. EuroIntervention : journal of EuroPCR in collaboration with the Working Group on Interventional Cardiology of the European Society of Cardiology. 2013;9(1):75-83.

30. Zhang Y, Shen J, Li Z, Zhu A, Yuan Y, Yue R, et al. Two-year clinical outcomes of different drug-eluting stents with different polymer coating strategies in coronary artery heart disease: a multi-centre, randomised, controlled clinical trial. International journal of cardiology. 2013;168(3):2646-52. Epub 2013/04/17. doi: 10.1016/j.ijcard.2013.03.034. PubMed PMID: 23587397.

31. Chen SL, Ye F, Zhang JJ, Zou JJ, Qian XS, Li F, et al. Real polymer-free sirolimus- and probucol-eluting versus biodegradable polymer sirolimus-eluting stents for obstructive coronary artery disease: DKPLUS-Wave 1, a multicenter, randomized, prospective trial. Cardiovascular therapeutics. 2013;31(4):193-200.

32. Smits PC, Hofma S, Togni M, Vazquez N, Valdes M, Voudris V, et al. Abluminal biodegradable polymer biolimus-eluting stent versus durable polymer everolimus-eluting stent (COMPARE II): A randomised, controlled, non-inferiority trial. The Lancet. 2013;381(9867):651-60.

33. Vlachojannis GJ, Smits PC, Hofma SH, Togni M, Vazquez N, Valdes M, et al. Long-term clinical outcomes of biodegradable polymer biolimus-eluting stents versus durable polymer everolimus-eluting stents in patients with coronary artery disease: three-year follow-up of the COMPARE II (Abluminal biodegradable polymer biolimus-eluting stent versus durable polymer everolimus-eluting stent) trial. EuroIntervention : journal of EuroPCR in collaboration with the Working Group on Interventional Cardiology of the European Society of Cardiology. 2015;11(3):272-9. Epub 2015/07/22. doi: 10.4244/eijv11i3a53. PubMed PMID: 26196753.

34. Natsuaki M, Kozuma K, Morimoto T, Kadota K, Muramatsu T, Nakagawa Y, et al. Biodegradable polymer biolimus-eluting stent versus durable polymer everolimus-eluting stent: A randomized, controlled, noninferiority trial. Journal of the American College of Cardiology. 2013;62(3):181-90.

35. Christiansen EH, Jensen LO, Thayssen P, Tilsted HH, Krusell LR, Hansen KN, et al. Biolimus-eluting biodegradable polymer-coated stent versus durable polymer-coated sirolimus-eluting stent in unselected patients receiving percutaneous coronary intervention (SORT OUT V): A randomised non-inferiority trial. The Lancet. 2013;381(9867):661-9.

36. Li Q, Tong Z, Wang L, Zhang J, Ge Y, Wang H, et al. Efficacy and safety of a biodegradable polymer sirolimus-eluting stent in primary percutaneous coronary intervention: A randomized controlled trial. Archives of Medical Science. 2013;9(6):1040-8.

37. Ribeiro EE, Campos CM, Ribeiro HB, Lopes AC, Esper RB, Meirelles GX, et al. First-in-man randomised comparison of a novel sirolimus-eluting stent with abluminal biodegradable polymer and thin-strut cobalt-chromium alloy: INSPIRON-I trial. EuroIntervention : journal of EuroPCR in collaboration with the Working Group on Interventional Cardiology of the European Society of Cardiology. 2014;9(12):1380-4.

38. Pilgrim T, Heg D, Roffi M, Tuller D, Muller O, Vuilliomenet A, et al. Ultrathin strut biodegradable polymer sirolimus-eluting stent versus durable polymer everolimus-eluting stent for percutaneous coronary revascularisation (BIOSCIENCE): a randomised, single-blind, non-inferiority trial. The Lancet. 2014.

39. Yuan F, Chen X, Song X, Wang D, Zhang Z, Li W, et al. Novel completed biodegradable polymer sirolimus-eluting stent versus durable polymer sirolimus-eluting stent in de novo lesions: nine-month angiographic and three-year clinical outcomes of HOPE trial. Chinese medical journal. 2014;127(14):2561-6. Epub 2014/07/22. PubMed PMID: 25043067.

40. Han Y, Xu B, Jing Q, Lu S, Yang L, Xu K, et al. A randomized comparison of novel biodegradable polymer-and durable polymer-coated cobalt-chromium sirolimus-eluting stents. JACC: Cardiovascular Interventions. 2014;7(12):1352-60.

41. Lee JY, Park DW, Kim YH, Ahn JM, Kim WJ, Kang SJ, et al. Comparison of biolimus A9-eluting (Nobori) and everolimus-eluting (Promus Element) stents in patients with de novo native long coronary artery lesions: A randomized long drug-eluting stent v trial. Circulation: Cardiovascular Interventions. 2014;7(3):322-9.

42. Zhang Q, Qiu JP, Kirtane AJ, Zhu TQ, Zhang RY, Yang ZK, et al. Comparison of biodegradable polymer versus durable polymer sirolimus-eluting stenting in patients with acute st-elevation myocardial infarction undergoing primary percutaneous coronary intervention: Results of the RESOLVE study. Journal of interventional cardiology. 2014;27(2):131-41.

43. Saito S, Valdes-Chavarri M, Richardt G, Moreno R, Iniguez Romo A, Barbato E, et al. A randomized, prospective, intercontinental evaluation of a bioresorbable polymer sirolimus-eluting coronary stent system: the CENTURY II (Clinical Evaluation of New Terumo Drug-Eluting Coronary Stent System in the Treatment of Patients with Coronary Artery Disease) trial. European heart journal. 2014;35(30):2021-31. Epub 2014/05/23. doi: 10.1093/eurheartj/ehu210. PubMed PMID: 24847155; PubMed Central PMCID: PMCPmc4200026.

44. Wijns W, Vrolix M, Verheye S, Schoors D, Slagboom T, Gosselink M, et al. Randomised study of a bioabsorbable polymer-coated sirolimus-eluting stent: results of the DESSOLVE II trial. EuroIntervention : journal of EuroPCR in collaboration with the Working Group on Interventional Cardiology of the European Society of Cardiology. 2015;10(12):1383-90. doi: 10.4244/EIJY14M05_03. PubMed PMID: 24801119.

45. Raungaard B, Jensen LO, Tilsted HH, Christiansen EH, Maeng M, Terkelsen CJ, et al. Zotarolimus-eluting durable-polymer-coated stent versus a biolimus-eluting biodegradable-polymer-coated stent in unselected patients undergoing percutaneous coronary intervention (SORT OUT VI): a randomised non-inferiority trial. Lancet (London, England). 2015;385(9977):1527-35. Epub 2015/01/21. doi: 10.1016/s0140-6736(14)61794-3. PubMed PMID: 25601789.

46. Puricel S, Arroyo D, Corpataux N, Baeriswyl G, Lehmann S, Kallinikou Z, et al. Comparison of everolimus- and biolimus-eluting coronary stents with everolimus-eluting bioresorbable vascular scaffolds. Journal of the American College of Cardiology. 2015;65(8):791-801. doi: 10.1016/j.jacc.2014.12.017. PubMed PMID: 25720622.

47. Kaiser C, Galatius S, Jeger R, Gilgen N, Skov Jensen J, Naber C, et al. Long-term efficacy and safety of biodegradable-polymer biolimus-eluting stents: main results of the Basel Stent Kosten-Effektivitats Trial-PROspective Validation Examination II (BASKET-PROVE II), a randomized, controlled noninferiority 2-year outcome trial. Circulation. 2015;131(1):74-81. Epub 2014/11/21. doi: 10.1161/circulationaha.114.013520. PubMed PMID: 25411159.

48. Shen L, Yang W, Yin JS, Liu XB, Wu YZ, Sun AJ, et al. Nine-month angiographic and two-year clinical follow-up of novel biodegradable-lpolymer arsenic trioxide-eluting stent versus durable-polymer sirolimus-eluting stent for coronary artery disease. Chinese medical journal. 2015;128(6):768-73.

49. Windecker S, Haude M, Neumann FJ, Stangl K, Witzenbichler B, Slagboom T, et al. Comparison of a novel biodegradable polymer sirolimus-eluting stent with a durable polymer everolimus-eluting stent: Results of the randomized BIOFLOW-II trial. Circulation: Cardiovascular Interventions. 2015;8(2).
