# Supplementary material for: Can Platforms Affect the Safety and Efficacy of Drug-Eluting Stents in the Era of Biodegradable Polymers?: A Meta-Analysis of 34,850 Randomized Individuals
Source: PLoS One. 2016 Mar 31;11(3):e0151259. doi: 10.1371/journal.pone.0151259 (PMC4816558; doi:10.1371/journal.pone.0151259)
Supplement: S4 Table — (DOC) [file pone.0151259.s007.doc]

**S4 Table. Definite/probable stent thrombosis (ST)**

|  | Maximum length of follow up(pooled ST)  **OR (95% CI)** | Within 30 days(early ST)  **OR (95% CI)** | Within 24h(acute ST)  **OR (95% CI)** | ＞24h-30 days(subacute ST)  **OR (95% CI)** | ＞30 days-1 year(late ST)  **OR (95% CI)** | Within 1 year(mid-term ST)  **OR (95% CI)** | ＞1 yea(long-term ST)  **OR (95% CI)** | Very late ST  **OR (95% CI)** |
| --- | --- | --- | --- | --- | --- | --- | --- | --- |
| BP-DESs vs other stents | **0.76(0.63,0.93)** | - | - | - | - | - | **-** | - |
| BP-stainless DESs vs other stents | **0.76(0.61,0.95)** | 0.93(0.62,1.41) | 1.37(0.26,7.29) | 1.22(0.28,5.31) | 0.67(0.38,1.17) | 0.82(0.63,1.06) | **0.73(0.57,0.94)** | **0.56(0.33,0.93)** |
| BP-stainless DESs vs other stainless DESs | 0.79(0.58,1.07) | 1.06(0.58,1.92) | - | - | 0.74(0.31,1.79) | 1.06(0.70,1.61) | **0.70(0.50,0.98)** | **0.29(0.14,0.58)** |
| BP-stainless DESs vs other alloy DESs | 0.74(0.50,1.09) | 0.82(0.46,1.46) | 1.37(0.26,7.29) | 1.22(0.28,5.31) | **0.46(0.22,0.98)** | 0.69(0.47,1.03) | 0.79(0.49,1.29) | 1.23(0.44,3.42) |
| BP-stainless DESs vs BMSs | 0.76(0.44,1.31) | - | - | - | - | 0.69(0.38,1.27) | 0.76(0.44,1.31) | 0.51(0.03,8.68) |
| BP-alloy DESs vs other stents | 0.76(0.51,1.15) | 0.81(0.48,1.37) | 0.52(0.11,2.50) | 1.06(0.30,3.75) | 0.71(0.35,1.43) | 0.79(0.52,1.20) | 0.23(0.04,1.39) | 0.33(0.05,2.12) |
| BP-alloy DESs vs other stainless DESs | 0.20(0.02,1.70) | - | - | - | 0.33(0.03,3.16) | 0.33(0.03,3.16) | 0.20(0.02,1.70) | 0.33(0.03,3.21) |
| BP-alloy DESs vs other alloy DESs | 0.80(0.53,1.22) | 0.81(0.48,1.37) | 0.52(0.11,2.50) | 1.06(0.30,3.75) | 0.77(0.37,1.60) | 0.81(0.53,1.24) | - | - |
| BP-alloy DESs vs BMSs | - | - | - | - | - | - | - | - |

BP indicates biodegradable polymer; DESs indicates drug-eluting stents; BMSs indicates bare metal stents; ‘-’ indicates not available.
