# Supplementary material for: Can Platforms Affect the Safety and Efficacy of Drug-Eluting Stents in the Era of Biodegradable Polymers?: A Meta-Analysis of 34,850 Randomized Individuals
Source: PLoS One. 2016 Mar 31;11(3):e0151259. doi: 10.1371/journal.pone.0151259 (PMC4816558; doi:10.1371/journal.pone.0151259)
Supplement: S5 Table — (DOCX) [file pone.0151259.s008.docx]

**S5 Table. Definite stent thrombosis (ST)**

|  | Maximum length of follow up(pooled ST)  OR (95% CI) | Within 30 days(early ST)  OR (95% CI) | Within 24h(acute ST)  OR (95% CI) | ＞24h-30 days(subacute ST)  OR (95% CI) | ＞30 days-1 year(late ST)  OR (95% CI) | Within 1 year(mid-term ST)  OR (95% CI) | ＞1 yea(long-term ST)  OR (95% CI) | Very late ST  OR (95% CI) |
| --- | --- | --- | --- | --- | --- | --- | --- | --- |
| BP-DESs vs other stents | 0.84(0.64,1.10) | - | - | - | - | - | - | - |
| BP-stainless DESs vs other stents | 0.83(0.61,1.13) | 1.28(0.75,2.18) | 1.60(0.38,6.71) | 1.69(0.37,7.71) | 0.86(0.45,1.66) | 0.96(0.66,1.40) | 0.71(0.53,0.96) | 0.65(0.29,1.44) |
| BP-stainless DESs vs other stainless DESs | 0.81(0.51,1.30) | 1.36(0.55,3.37) | - | - | 0.46(0.16,1.30) | 0.96(0.51,1.83) | 0.63(0.43,0.93) | 0.24(0.10,0.59) |
| BP-stainless DESs vs other alloy DESs | 1.08(0.65,1.81) | 1.32(0.56,3.08) | 0.70(0.11,4.47) | 1.20(0.16,8.74) | 1.21(0.48,3.04) | 1.20(0.65,2.19) | 1.09(0.58,2.04) | 1.38(0.41,4.73) |
| BP-stainless DESs vs BMSs | 0.62(0.30,1.27) | - | - | - | - | 0.56(0.22,1.45) | 0.62(0.30,1.27) | 1.55(0.38,6.34) |
| BP-alloy DESs vs other stents | 0.94(0.46,1.92) | 1.12(0.34,3.63) | - | - | 1.49(0.34,6.50) | 0.96(0.43, 2.15) | - | - |
| BP-alloy DESs vs other stainless DESs | - | - | - | - | - | - | - | - |
| BP-alloy DESs vs other alloy DESs | 0.96(0.43,2.15) | 1.12(0.34,3.63) | - | - | 1.49(0.34,6.50) | 0.96(0.43,2.15) | - | - |
| BP-alloy DESs vs BMSs | - | - | - | - | - | - | - | - |

BP indicates biodegradable polymer; DESs indicates drug-eluting stents; BMSs indicates bare metal stents; ‘-’ indicates not available
