# Supplementary material for: Can Platforms Affect the Safety and Efficacy of Drug-Eluting Stents in the Era of Biodegradable Polymers?: A Meta-Analysis of 34,850 Randomized Individuals
Source: PLoS One. 2016 Mar 31;11(3):e0151259. doi: 10.1371/journal.pone.0151259 (PMC4816558; doi:10.1371/journal.pone.0151259)
Supplement: S6 Table — (DOC) [file pone.0151259.s009.doc]

**S6 Table. Death**

|  | Maximum length of follow up(pooled)  **OR (95% CI)** | Within 30 days(short-term)  **OR (95% CI)** | Within 1 year(mid-term)  **OR (95% CI)** | ＞1 year(long-term)  **OR (95% CI)** |
| --- | --- | --- | --- | --- |
| BP-DESs vs other stents | 0.93(0.84,1.03) | - | - | - |
| BP-stainless DESs vs other stents | 0.92(0.82,1.02) | 0.90(0.54,1.5) | 0.96(0.82,1.12) | 0.91(0.81,1.04) |
| BP-stainless DESs vs other stainless DESs | 0.86(0.73,1.02) | 0.82(0.41,1.64) | 0.99(0.72,1.37) | **0.84(0.71,1.00)** |
| BP-stainless DESs vs other alloy DESs | 0.99(0.84,1.16) | 1.01(0.47,2.18) | 0.94(0.74,1.18) | 1.05(0.85,1.29) |
| BP-stainless DESs vs BMSs | 0.83(0.57,1.23) | - | 0.88(0.49,1.59) | 0.82(0.56,1.22) |
| BP-alloy DESs vs other stents | 1.07(0.77,1.48) | 1.85(0.36,9.58) | 1.01(0.72,1.41) | 1.10(0.57,2.13) |
| BP-alloy DESs vs other stainless DESs | 0.74(0.36,1.52) | - | 0.52(0.21,1.31) | 0.76(0.31,1.86) |
| BP-alloy DESs vs other alloy DESs | 1.20(0.83,1.73) | 1.77(0.32,9.56) | 1.18(0.81,1.72) | 2.18(0.15,31.72) |
| BP-alloy DESs vs BMSs | 0.52(0.06,4.77) | - | - | - |

BP indicates biodegradable polymer; DESs indicates drug-eluting stents; BMSs indicates bare metal stents; ‘-’ indicates not available.
