# Supplementary material for: Can Platforms Affect the Safety and Efficacy of Drug-Eluting Stents in the Era of Biodegradable Polymers?: A Meta-Analysis of 34,850 Randomized Individuals
Source: PLoS One. 2016 Mar 31;11(3):e0151259. doi: 10.1371/journal.pone.0151259 (PMC4816558; doi:10.1371/journal.pone.0151259)
Supplement: S7 Table — (DOC) [file pone.0151259.s010.doc]

**S7 Table. Cardiac death**

|  | Maximum length of follow up(pooled)  **OR (95% CI)** | Within 30 days(short-term)  **OR (95% CI)** | ＞30 days-1 year(mid-term)  **OR (95% CI)** | ＞1 year(long-term)  **OR (95% CI)** |
| --- | --- | --- | --- | --- |
| BP-DESs vs other stents | 0.96(0.83,1.10) | - | - | - |
| BP-stainless DESs vs other stents | 0.96(0.83,1.12) | 0.81(0.52,1.26) | 0.96(0.80,1.17) | 0.93(0.78,1.11) |
| BP-stainless DESs vs other stainless DESs | 0.89(0.71,1.12) | 0.78(0.37,1.64) | 0.84(0.55,1.27) | 0.90(0.70,1.15) |
| BP-stainless DESs vs other alloy DESs | 1.08(0.87,1.33) | 1.11(0.47,2.62) | 1.11(0.84,1.46) | 1.03(0.76,1.39) |
| BP-stainless DESs vs BMSs | 0.87(0.44,1.73) | - | 0.85(0.50,1.47) | 0.87(0.44,1.73) |
| BP-alloy DESs vs other stents | 0.93(0.60,1.43) | - | 0.91(0.59,1.41) | 0.64(0.21,1.96) |
| BP-alloy DESs vs other stainless DESs | 0.35(0.04,2.93) | - | - | 0.35(0.04,2.93) |
| BP-alloy DESs vs other alloy DESs | 1.00(0.64,1.56) | - | 0.97(0.61,1.54) | 1.48(0.23,9.45) |
| BP-alloy DESs vs BMSs | - | - | - | - |

BP indicates biodegradable polymer; DESs indicates drug-eluting stents; BMSs indicates bare metal stents; ‘-’ indicates not available.
