# Supplementary material for: Can Platforms Affect the Safety and Efficacy of Drug-Eluting Stents in the Era of Biodegradable Polymers?: A Meta-Analysis of 34,850 Randomized Individuals
Source: PLoS One. 2016 Mar 31;11(3):e0151259. doi: 10.1371/journal.pone.0151259 (PMC4816558; doi:10.1371/journal.pone.0151259)
Supplement: S8 Table — (DOC) [file pone.0151259.s011.doc]

**S8 Table. Myocardial infarction**

|  | Maximum length of follow up(pooled)  **OR (95% CI)** | Within 30 days(short-term)  **OR (95% CI)** | ＞30 days-1 year(mid-term)  **OR (95% CI)** | ＞1 year(long-term)  **OR (95% CI)** |
| --- | --- | --- | --- | --- |
| BP-DESs vs other stents | 0.98(0.88,1.09) | - | - | - |
| BP-stainless DESs vs other stents | 0.98(0.86,1.11) | 1.10(0.87,1.39) | 0.99(0.85,1.14) | 0.95(0.81,1.12) |
| BP-stainless DESs vs other stainless DESs | 1.01(0.83,1.24) | 1.21(0.74,1.98) | 1.22(0.91,1.65) | 0.95(0.76,1.20) |
| BP-stainless DESs vs other alloy DESs | 1.01(0.85,1.20) | 0.90(0.59,1.39) | 0.96(0.77,1.18) | 1.07(0.83,1.39) |
| BP-stainless DESs vs BMSs | 0.69(0.45,1.06) | - | **0.60(0.38,0.95)** | 0.69(0.45,1.06) |
| BP-alloy DESs vs other stents | 0.98(0.78,1.22) | 1.04(0.76,1.41) | 0.99(0.79,1.24) | 1.21(0.61,2.42) |
| BP-alloy DESs vs other stainless DESs | 1.21(0.72,2.02) | - | 1.26(074,2.16) | 0.69(0.26,1.84) |
| BP-alloy DESs vs other alloy DESs | 0.92(0.72,1.18) | 0.90(0.63,1.27) | 0.91(0.70,1.17) | 2.23(0.54,9.30) |
| BP-alloy DESs vs BMSs | - | - | - | - |

BP indicates biodegradable polymer; DESs indicates drug-eluting stents; BMSs indicates bare metal stents; ‘-’ indicates not available.
