# Supplementary material for: Can Platforms Affect the Safety and Efficacy of Drug-Eluting Stents in the Era of Biodegradable Polymers?: A Meta-Analysis of 34,850 Randomized Individuals
Source: PLoS One. 2016 Mar 31;11(3):e0151259. doi: 10.1371/journal.pone.0151259 (PMC4816558; doi:10.1371/journal.pone.0151259)
Supplement: S9 Table — (DOC) [file pone.0151259.s012.doc]

**S9 Table. Target lesion revascularization**

|  | Maximum length of follow up(pooled)  **OR (95% CI)** | Within 30 days(short-term)  **OR (95% CI)** | ＞30 days-1 year(mid-term)  **OR (95% CI)** | ＞1 year(long-term)  **OR (95% CI)** |
| --- | --- | --- | --- | --- |
| BP-DESs vs other stents | **0.74(0.62,0.89)** |  |  |  |
| BP-stainless DESs vs other stents | **0.73(0.59,0.91)** | 0.91(0.62,1.34) | **0.68(0.53,0.87)** | **0.72(0.55,0.94)** |
| BP-stainless DESs vs other stainless DESs | **0.73(0.54,1.00)** | 0.83(0.36,1.91) | **0.58(0.35,0.97)** | **0.80(0.67,0.95)** |
| BP-stainless DESs vs other alloy DESs | 1.00(0.81,1.22) | 1.13(0.57,2.23) | 0.93(0.73,1.19) | 1.11(0.87,1.43) |
| BP-stainless DESs vs BMSs | **0.33(0.23,0.49)** | - | **0.29(0.17,0.49)** | **0.32(0.22,0.48)** |
| BP-alloy DESs vs other stents | 0.76(0.53,1.11) | 0.48(0.12,1.89) | 0.88(0.66,1.18) | 0.45(0.18,1.11) |
| BP-alloy DESs vs other stainless DESs | 0.87(0.22,3.50) | - | 0.90(0.28,2.88) | 0.87(0.22,3.50) |
| BP-alloy DESs vs other alloy DESs | 0.99(0.72,1.36) | 0.53(0.07,3.83) | 1.07(0.80,1.43) | 0.35(0.12,1.02) |
| BP-alloy DESs vs BMSs | 0.23(0.03,2.11) | - | - | - |

BP indicates biodegradable polymer; DESs indicates drug-eluting stents; BMSs indicates bare metal stents; ‘-’ indicates not available.
