# Supplementary material for: Can Platforms Affect the Safety and Efficacy of Drug-Eluting Stents in the Era of Biodegradable Polymers?: A Meta-Analysis of 34,850 Randomized Individuals
Source: PLoS One. 2016 Mar 31;11(3):e0151259. doi: 10.1371/journal.pone.0151259 (PMC4816558; doi:10.1371/journal.pone.0151259)
Supplement: S10 Table — (DOC) [file pone.0151259.s013.doc]

**S10 Table. Target vessel revascularization**

|  | Maximum length of follow up(pooled)  **OR (95% CI)** | Within 30 days(short-term)  **OR (95% CI)** | ＞30 days-1 year(mid-term)  **OR (95% CI)** | ＞1 year(long-term)  **OR (95% CI)** |
| --- | --- | --- | --- | --- |
| BP-DESs vs other stents | **0.76(0.62,0.93)** | - | - | - |
| BP-stainless DESs vs other stents | **0.74(0.58,0.96)** | 0.89(0.51,1.55) | **0.72(0.55,0.94)** | **0.63(0.44,0.91)** |
| BP-stainless DESs vs other stainless DESs | 0.93(0.65,1.33) | 0.96(0.31,2.94) | 0.84(0.52,1.35) | 0.86(0.68,1.08) |
| BP-stainless DESs vs other alloy DESs | 1.09(0.92,1.28) | 0.82(0.43,1.54) | 1.10(0.91,1.31) | 1.15(0.89,1.49) |
| BP-stainless DESs vs BMSs | **0.34(0.22,0.52)** | - | **0.29(0.20,0.41)** | **0.34(0.22,0.52)** |
| BP-alloy DESs vs other stents | 0.83(0.61,1.13) | **0.22(0.05,0.95)** | 0.93(0.72,1.21) | **0.31(0.14,0.68)** |
| BP-alloy DESs vs other stainless DESs | - | - | - | - |
| BP-alloy DESs vs other alloy DESs | 0.97(0.71,1.33) | - | 1.09(0.85,1.39) | **0.34(0.14,0.81)** |
| BP-alloy DESs vs BMSs | **0.50(0.28,0.92)** | - | - | - |

BP indicates biodegradable polymer; DESs indicates drug-eluting stents; BMSs indicates bare metal stents; ‘-’ indicates not available.
